# Prevalence and association of anxiety and depression among orthopaedic trauma inpatients: a retrospective analysis of 1994 cases

**DOI:** 10.1186/s13018-020-02132-4

**Published:** 2020-12-07

**Authors:** Yun Yang, Ting-ting Tang, Mei-ru Chen, Mao-ying Xiang, Ling-li Li, Xiao-ling Hou

**Affiliations:** 1 Department of Orthopaedics, West China Hospital, Sichuan University, Chengdu, Sichuan People’s Republic of China; 2 School of Nursing, West China Hospital of Sichuan University, Chengdu, Sichuan People’s Republic of China

**Keywords:** Anxiety, Depression, Prevalence, Risk factors, Orthopaedic trauma

## Abstract

**Background:**

Patients with traumatic injuries are often accompanied by emotional disorders, which seriously impede functional gains. The objective of this study was to identify the prevalence and risk factors associated with underlying anxiety and depression in orthopaedic trauma patients.

**Methods:**

From July 2015 to December 2017, all orthopaedic trauma patients were included in the retrospective study. Patients with conditions that might affect cognitive impairment were excluded from the study. Basic demographic data were collected. All patients were screened for emotional disorders on admission using a simple questionnaire called “Huaxi Emotional-Distress Index” (HEI). Bivariate analyses and logistic regression were used to identify the factors associated with a HEI score of > 8.

**Results:**

One hundred and sixty-two patients (8.1%) had a HEI score of > 8. About 1.0% of enrolled patients had severe emotional disorders (HEI score ≥ 17). The reasons caused by emotional disorders in patients with orthopaedic trauma were a higher Injury Severity Score (ISS), a higher visual analogue score (VAS) and type of surgery. On logistic regression, marital status was a protective factor for emotional disorders, while VAS and ISS were the risk factors for emotional disorders.

**Conclusions:**

Although a significantly low percentage of orthopaedic trauma patients in our setting have emotional disorders, traumatic orthopaedic surgeons still need to pay attention to the risk of emotional disorders and integrate effective screening tools into clinical practice to screen for these factors and stratify emotional disorders. Appropriate targeted psychological intervention and treatment should be adopted according to the stratification of emotional disorders.

## Background

With the improvement of surgical techniques and the development of implants, significant advancements have been made in the management of orthopaedic trauma patients. However, orthopaedic surgeons tend to focus on physical and technical factors in the treatment of these musculoskeletal injuries. In fact, drastic physical changes, strange hospital environment and uncertainty of post-injury recovery affect patients’ psychology. Anxiety and depression in orthopaedic trauma patients have been reported to range between 5–35% and 13–56%, respectively [1–3]. Some studies have found that emotional disorder (anxiety or depression) was associated with factors such as pain, nuclear family, female sex [4], severity of injury [5], a younger age, lack of social support and use of cannabis [6, 7]. Several studies have established these psychological factors adversely affect the outcomes [8–10]. Therefore, it is of great significance to understand and master the psychological characteristics and related factors of orthopaedic trauma patients in order to promote rapid rehabilitation.

At present, while there are many scales for screening anxiety, depression and negative emotions [11–14], few scales can screen these three aspects at the same time. In addition, these scales are rarely used in routine clinical practice due to their disadvantages, which include too many items, time consumption and cultural or language barriers. Therefore, based on the Chinese population and culture, Wang et al. [15] developed a new psychometrically solid and concise screening tool for identifying emotional disorders (anxiety, depression and/or suicidal ideation). The questionnaire was named as “Huaxi Emotional-Distress Index (HEI)”. The Cronbach’s *α* of HEI was 0.90; sensitivity and specificity were 0.880 and 0.766, respectively. The HEI has shown good effectiveness when used in patients or medical staff, which is characterized by fewer items, less time and easy accessibility [15, 16].

Early detection of emotional disorders in patients is crucial as it gives medical staff a chance to further improve and promote clinical medical treatment and nursing work. Therefore, this study’s aim was to determine the prevalence of underlying emotional disorders among orthopaedic trauma inpatients and identify related factors at a level 1 trauma centre.

## Materials and methods

### Subjects

A retrospective evaluation was conducted of orthopaedic trauma patients between July 2015 and December 2017. The inclusion criteria were as follows: (1) age greater than or equal to 15 years and (2) musculoskeletal injuries (including fracture, joint dislocation and soft tissue injuries of nerve, muscle, blood vessel, etc.). Patients with conditions that might affect cognitive impairment were excluded from the study. These included conditions such as head injury, chronic neurological illness, history of psychiatric disorder, chronic ongoing illness and intellectual disability. The questionnaire was conducted only when patients reported physical comfort. Data were collected through an anonymous way because the patients’ identifiers such as name and unique identity were erased. Before the study began, we had carefully consulted the Ethics Committee and Institutional Review Board of West China Hospital. They suggested that this study did not involve special interventions for patients and we should conduct this study in compliance with the Helsinki Declaration. So, all data was fully anonymised at source. Given the anonymous nature of the data, the ethics committee waived any requirement for patient informed consent.

### Huaxi emotional-distress index

The HEI is a preliminary screening tool for emotional disorders (anxiety and depression) in non-psychiatric clinical settings [15]. It is mainly used for rapid screening and grading of emotional disorders and related mental health problems. There are 9 self-reported items in total that can be finished in less than 5 min. All items are 5-point Likert-scaled with scale points 0, 1, 2, 3 and 4. The total score is the sum of the scores of 9 items. There are four grades based on the total score: normal (0–8 points), mild (9–12 points), moderate (13–16 points) and severe (17–36 points). If there is a relatively serious emotional disorder, it will automatically expand to 11 items. The last 2 additional items are not included in the score, but the results are for the reference of clinical workers. Details of the HEI are shown in Table 1.

**Table 1:**
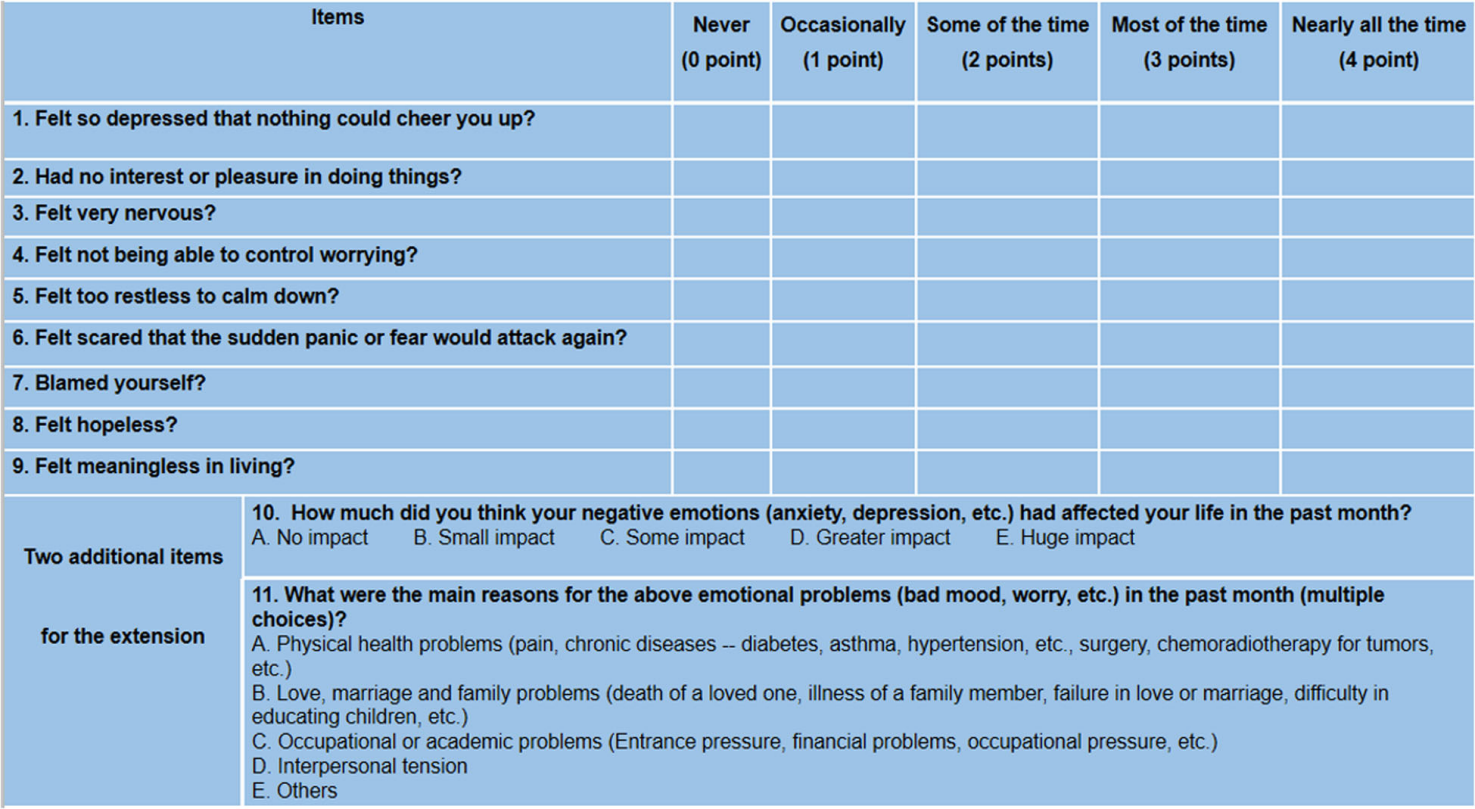
Huaxi Emotional-Distress Index (HEI)

### Assessment method

**Fig. 1:**
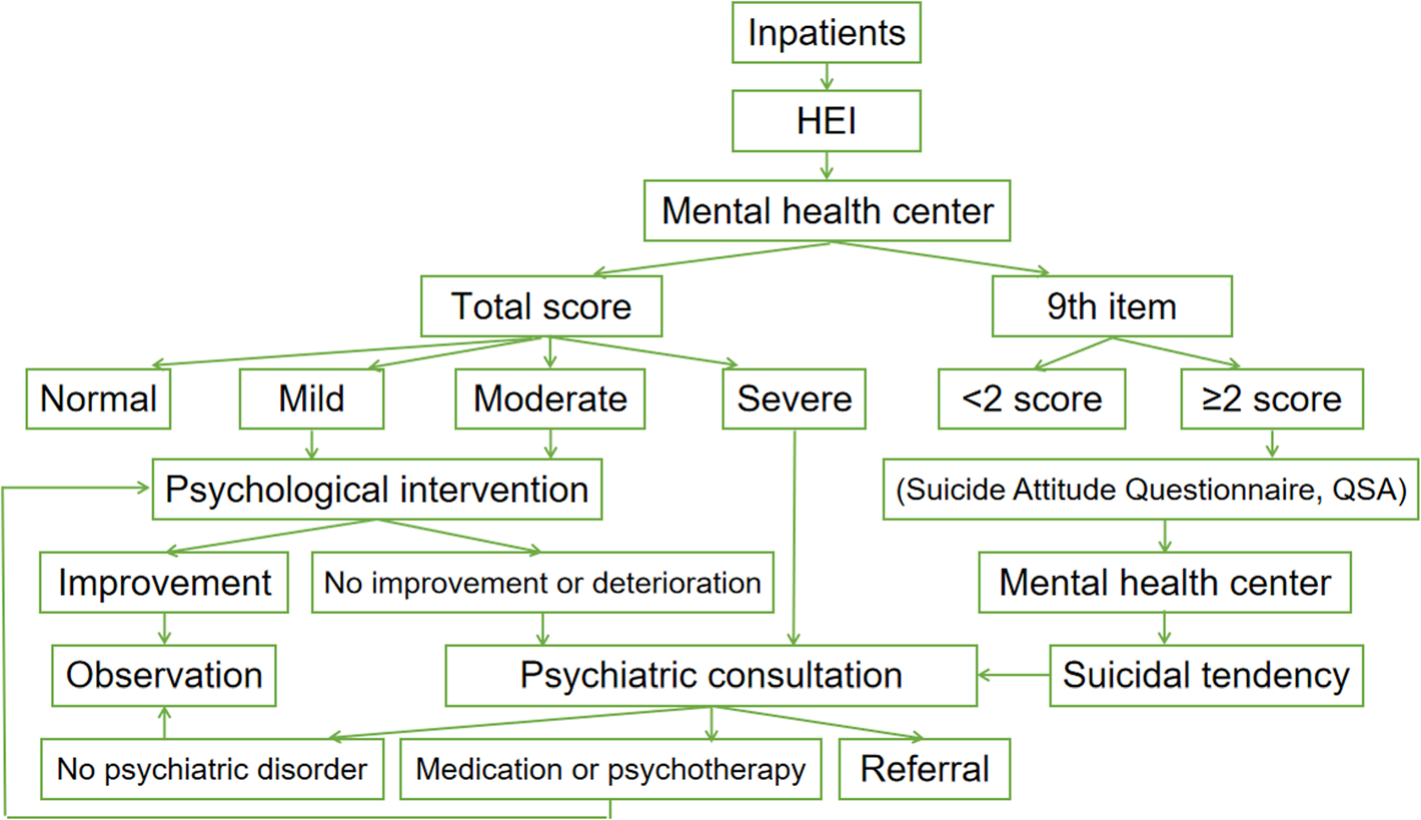
The process of HEI assessment and coping strategies

Generally, the paper version of the questionnaire was sent to patients, who filled in the results according to their actual situation. If the patient had difficulty completing the questionnaire, such as a hand injury, the nurse staff would help fill in the questionnaire according to their dictation. Then, the nursing staff uploaded the results filled out by the patient to the HIS system, and the doctors of mental health centre checked the patient’s filling results through the HIS system and timely feedbacked the report. For patients with mild to moderate emotional disorder, psychological counselling should be conducted first by nurses who have received psychological training and obtained certificates. When necessary, psychiatric consultation was conducted for specialized treatment. A more systematic suicide risk assessment was required if the score of the 9th item was equal to or higher than 2 or if the score indicated a severe emotional disorder and the score was verified to reflect the patient’s true feelings. For these patients, psychiatric consultation and specialized treatment were the first steps. Secondly, a weekly questionnaire was required during the hospitalization. The detailed process was shown in Fig. 1.

### Statistical analysis

Statistical analysis of the data was performed using the SPSS 20.0 software (SPSS Chicago, IL, USA). The statistical methods adopted included frequency, percentage (%), mean ± standard, *t* test, univariate analysis, correlation analysis and multiple linear regression analysis. A value of *p* ≤ 0.05 was considered to be statistically significant.

## Results

According to the inclusion and exclusion criteria, 1994 patients were included in the study. The age ranged from 15 to 90, with an average age of 47.8 ± 17.5. Nearly two thirds of the patients were males. The vast majority (87.71%) of patients were admitted to hospital with fractures. The average Injury Severity Score (ISS) was 8.0 ± 4.4. Nearly 75% of the patients had a high school degree or less. Nearly a third of the patients (34.7%) required emergency surgery. Baseline data of the enrolled patients were shown in Table 2.

**Table 2:** The baseline data of the enrolled patients

Variables	
**Age (years)**	
≤ 29	360 (18.06%)
30–49	753 (37.76%)
≥ 50	881 (44.18%)
**Gender**	
Male	1317 (66.05%)
Female	677 (33.95%)
**Injury Severity Score (ISS)**	7.97 ± 4.44
**Mean pain score at admission (VAS)**	6.52 ± 1.24
**Types of musculoskeletal injury**	
Fracture	1749 (87.71%)
Joint dislocation	99 (4.97%)
Soft tissue injuries	146 (7.32%)
**Educational level**	
Primary school or below	618 (30.99%)
High school	892 (44.73%)
Junior college	254 (12.74%)
Undergraduate	173 (8.68%)
Graduate or higher	25 (1.25%)
Others	32 (1.61%)
**Marital status**	
Married	1603 (80.39%)
Unmarried	294 (14.75%)
Divorced or widowed	97 (4.86%)
**Surgery**	
Emergency	692 (34.70%)
Elective	1249 (62.64%)
None	53 (2.66%)
**HEI score**	
≤ 8	1832 (91.88%)
> 8	162 (8.12%)
**Total**	1994 (100%)

If joint dislocation and fracture occurred at the same time, it was considered joint dislocation. Junior college included college degree and technical secondary school. Others in educational level referred to patients who did not want to disclose their education

One hundred and sixty-two patients (8.1%) had a HEI score of > 8 (Table 2). Twenty patients had a score of ≥ 17, suggesting that about 1.0% of patients with orthopaedic trauma had severe emotional disorders. A higher ISS, a higher visual analogue score (VAS) and type of surgery were found to be significantly associated (*p* value < 0.05) with a HEI score of > 8 (Table 3). Because 32 patients did not want to disclose their educational levels, we treated these patients as missing values here (Table 4).

**Table 3:** Association between the level of HEI score and related factors

Variables	HEI score ≤ 8 (*n* (%))	HEI score > 8 (*n* (%))	*p* value
**Age (years)**	48.00 ± 17.44	45.84 ± 17.78	0.172
**Gender**			
Male	1212 (66.16%)	105 (64.81%)	0.729
Female	620 (33.84)	57 (35.19%)	
**Injury Severity Score (ISS)**	7.84 ± 4.42	9.54 ± 4.37	< 0.001
**Mean pain score at admission (VAS)**	6.48 ± 1.25	6.88 ± 1.12	< 0.001
**Types of musculoskeletal injuries**			
Fracture	1606 (87.67%)	143 (88.27%)	0.708
Joint dislocation	93 (5.07%)	6 (3.70%)	
Soft tissue injuries	133 (7.26)	13 (8.03)	
**Marital status**			
Married	1481 (80.84%)	14 (8.64%)	0.051
Unmarried	268 (14.63%)	26 (16.05%)	
Divorced or widowed	83 (4.53%)	122 (75.31%)	
**Surgery**			
Emergency	626 (34.17%)	66 (40.74%)	0.027
Elective	1161 (63.37%)	88 (54.32%)	
None	45 (2.46%)	8 (4.94%)

**Table 4:** Association of the level of education with HEI score > 8

	HEI score ≤ 8	HEI score > 8	*p* value
**Educational level**			
Primary school or below	574 (31.85%)	44 (27.50%)	0.936
High school	806 (44.73%)	86 (53.75%)	
Junior college	234 (12.99%)	20 (12.50%)	
Undergraduate	164 (9.10%)	9 (5.63%)	
Graduate or higher	24 (1.33%)	1 (0.62%)	
**Total**	1802 (100%)	160 (100%)	

Because 32 patients did not want to disclose their educational levels, we treated these patients as missing values here and did not include univariate analysis

Stepwise logistic regression was used to identify significant predictors of HEI > 8. Marital status was a protective factor for emotional disorders. Specifically, the risk of emotional disorders in married patients was 0.5 times that of unmarried patients, while divorced or widowed patients had a 0.4 times higher risk than unmarried patients. The VAS and ISS were risk factors for emotional disorders. The risk of emotional disorders increased by 1.2 times and 1.1 times, respectively, for every 1 point increase in these two factors (Table 5).

**Table 5:** Logistic regression analysis showing the relationship of significant emotional distress (anxiety and/or depression) predictors with HEI score > 8

	*B*	S.E.	*p* value	Exp (*B*)/OR
**Injury Severity Score (ISS)**	0.060	0.020	0.002	1.062
**Mean pain score at admission (VAS)**	0.162	0.076	0.034	1.175
**Marital status**			0.022	
Married	− 0.720	0.361	0.046	0.487
Divorced or widowed	− 0.855	0.310	0.006	0.425
**Constant**	− 3.239	0.544	0.000	0.039

## Discussion

Orthopaedic trauma is an unforeseen life-changing event [17] that can disrupt multiple dimensions of health-related quality of life. Nearly 2.8 million Americans sustain traumatic orthopaedic injuries annually [18]. In addition to limited physical activity, these patients are often interfered with social roles, pain and emotional disorders [19]. Although significant advancements have been made in the treatment of orthopaedic injuries [20, 21], high rates of disability following musculoskeletal injury remain prevalent. Current orthopaedic trauma research has been substantially focused on rapid stabilization of the patient and reconstruction of musculoskeletal tissues. Although physical trauma can be effectively treated, emotional disorders in orthopaedic trauma inpatients are often ignored and impede functional recovery [10]. A growing body of literature has pointed to the substantial importance of psychosocial factors in recovery from traumatic orthopaedic injuries [8–10]. Therefore, efficient screening and early identification of emotional disorders are helpful for orthopaedic trauma surgeons to take active and effective interventions to prevent adverse events, with a view to improving outcomes.

In our study, the frequency of emotional disorders (HEI score > 8) was 8.1%. Our result was much lower than the prevalence of emotional disorders (anxiety and depression) reported in other studies [4, 10, 22, 23]. This was likely due to the heterogeneity of the populations studied and the instruments used to measure these psychological parameters [24, 25]. In addition, there were some objective practical reasons that might explain the relatively low prevalence. On the one hand, many patients came to our hospital with high expectations of good physical function due to their great trust in our hospital. On the other hand, a significant number of patients were referred from other hospitals or lower-level hospitals and received initial treatment. At the same time, the data of the included cases in this study were all from the first questionnaire of the patients at the time of admission. Thus, those patients with no emotional disorders at the time of admission developed negative emotions during the hospitalization, and these patients were not included in the study. These reasons may result in a low prevalence of emotional disorders.

On logistic regression, we found the VAS, ISS and marital status to be associated with a HEI score > 8. A study of multivariable analysis reported that a higher pain score, nuclear family and female sex were associated with depression in orthopaedic trauma patients [4]. Another study reported that only open fractures had an effect on the presence of depression among injury-specific factors [10]. Giannoudis et al. [5] found that the presence of anxiety was related to the severity of injury in the lower extremity. Other studies found that a younger age, lack of social support and use of cannabis were positively correlated with depression [6, 7]. We did not find any association between age and sex and HEI score.

Pain has a detrimental effect on patients’ quality of life as it often leads to emotional disorders such as anxiety and depression. Although the link between depression and chronic pain has been well established [26], similar research on acute musculoskeletal pain is limited. We found a significant positive association between acute post-traumatic pain and HEI score > 8, which was consistent with the results reported in other literature [4, 27]. In our experience, patients were most likely to exhibit catastrophic thinking and became anxious or depressed when in pain, which in turn exacerbated the pain, leading to a vicious cycle. To this end, for orthopaedic trauma patients, it is necessary to actively manage pain to reduce anxiety or depression.

A few studies have found no relationship between depression and ISS [4, 10]. However, we found ISS to be significantly positively associated with HEI > 8 score. In this study, these patients with severe injuries had relatively complex conditions, such as open fractures or multiple fractures. These patients had long hospital stays, which contradicted their desire for good physical function, and were prone to negative emotions. For such patients, in addition to the first questionnaire at admission, a weekly questionnaire was often required during the hospitalization in order to timely find out potential negative emotions and deal with them accordingly.

Several studies have reported the relationship between social and family support and depression [6, 28]. In our study, marital status was found to be associated with HEI > 8 on logistic regression. The unmarried patients had a higher risk of emotional disorders than those married patients. The loss of family support resulted in physical and psychological isolation. Unmarried patients tended to have negative emotions because they felt isolated. A good family atmosphere was beneficial to the improvement of patients’ negative emotions, especially for those who were seriously damaged and needed more care and company from their families or partners. In this study, the patients might feel that their families, especially their children, provided them with increased social support after acute orthopaedic trauma, and the patients were satisfied with this support. This might be related to the importance attached to filial piety in traditional Chinese culture. These might well explain the low incidence of emotional disorders in this study.

This study has several limitations. First, some baseline characteristics (such as alcohol abuse, smoking, economic status) were not included in the study. Second, data were collected from a single level 1 trauma centre and may affect their representativeness and consistency. Third, this study did not include some other possible psychological variables, such as post-traumatic stress disorder common to orthopaedic trauma patients. Therefore, further research could expand the coverage and diversity of samples and increase layers of research design.

## Conclusion

This study uses a new psychometrically solid and concise screening tool that effectively and efficiently screens emotional disorders. In high-risk groups, such as patients with a higher ISS, higher pain score or being unmarried, it is especially important for providers to be aware of the possible negative effect of emotional disorders on patient outcomes. Although a significantly low percentage of orthopaedic trauma patients in our setting have emotional disorders, we recommend emotional screening and stratification for in-patient orthopaedic trauma patients. According to the stratification of emotional disorders, appropriate targeted psychological intervention and treatment should be adopted.

## Data Availability

Datasets are available from the corresponding author on reasonable request.

## Abbreviations

HEI: Huaxi Emotional-Distress Index
ISS: Injury Severity Score
VAS: Visual analogue scale/score
